# The Hydrobromates

**Published:** 1887-12-17

**Authors:** 


### THE HYDROBROMATES.

Hydrobromic Acid has for some time been used in "medicine." It possesses the same physiological action as the bromides ordinarily in use, though perhaps in a less marked degree. It dissolves very readily in water, and forms a clear solution with the sulphate of quinia and also with the perchloride of iron, or with the two together. Advantage has been taken of these facts by Messrs. Fletcher, Fletcher, and Stevenson, to produce syrups which give, it is said, all the effects of the bromides, quinine and iron, without any of their inconveniences. The sedative action of the bromides, the tonic action of quinine, the blood-making property of iron, and the nerve-restoring power of strychnia are well known, and need not be dwelt upon. If it be true that in a single syrup the properties of all these drugs may be combined, and made available for the patients, it is clear that a valuable substance is produced. Experience only can decide whether such a substance possesses the qualities claimed for it; and experience here seems quite to coincide with theoretical anticipation. Some people cannot take quinine, at any rate in sufficient quantities to be of any service to them. Very small doses produce all the unpleasant effects of marked cinchonism, ringing in the ears, suffused face, severe bursting headache, and other disagreeable and, to the patient, alarming symptoms. So much is this the case with certain persons—and these not a few—that nothing will induce them to take quinine if they know it. If now we are able to combine it with something which will entirely take away these unpleasant effects, we may often do a great service to people who stand in need of such tonic action as nothing but quinine can afford. Hydrobromic acid given with quinine, and forming with it a hydrobromate, is said to obviate entirely the tendency to cinchonism. How this is done it is impossible to say, because observers are not yet agreed whether quinine produces its effects on the brain by inducing excess or defect of blood; cerebral congestion or cerebral anæmia. If by producing cerebral anæmia, then the action of the hydrobromic acid seems not only difficult but impossible of explanation. But if by congestion, then the acid, by checking the tendency to congestion and merely allowing such an increased flow of blood as will cause improved nutrition and tone, exerts an action which is perfectly comprehensible. Be the physiological effect what it may, the principal point for the physician is to ascertain whether the hydrobromate of quinia can or can not be taken with advantage and comfort by those who find it impossible to take quinine in any other form. The makers say it can, and they adduce a large amount of medical testimony in favour of their contention. It seems highly probable that they are right. Fletcher's syrups have been for some years in the market, and have met with general approval. They are exceedingly pure and delicate preparations, and very convenient for use. Two syrups are principally employed in practice: the syrup of the hydrobromate of iron and quinine, and the syrup of the hydrobromate of iron, quinine, and strychnia. From a very frequent experience of their action, extending over four or five years, we are able to affirm that in many cases of debility, cerebral anæmia, nervous exhaustion resulting from overwork and poor assimilation of food and similar conditions, the use of these syrups has been followed by excellent results in a very short time. There is little doubt that they are genuine preparations of the drugs specified and of the strength stated; and their combination does not seem to have impaired the effect of any of the separate constituents but rather to have enhanced the efficacy of them all.

